# Noninvasive ventilation: A survey of practice patterns of its use in India

**DOI:** 10.4103/0972-5229.45076

**Published:** 2008

**Authors:** Rajesh Chawla, U. S. Sidhu, Vijai Kumar, Shruti Nagarkar, Laurent Brochard

**Affiliations:** **From:** Department of Pulmonary, Critical Care and Sleep Medicine, Indraprastha Apollo Hospitals, New Delhi, India; 1 Department of Pulmonary, Critical Care and Sleep Medicine, Dayanand Medical College, Ludhiana, India; 2 Department of Pulmonary, Critical Care and Sleep Medicine, Yashoda Superspeciality Hospital, Secundrabad, India; 3 Department of Pulmonary, Critical Care and Sleep Medicine, Bombay Hospital, Mumbai, India; 4 Department of Pulmonary, Critical Care and Sleep Medicine, Intensive Care Medicine, University of Paris, Paris, France

**Keywords:** Noninvasive ventilation, questionnaire-based study, survey

## Abstract

**Background and Aims:**

To understand the practice patterns of noninvasive ventilation (NIV) use by Indian physicians.

**Subjects and Methods:**

Around three thousand physicians from all over India were mailed a questionnaire that could capture the practice patterns of NIV use.

**Results:**

Completed responses were received from 648 physicians (21.6%). Majority (*n* = 469, 72.4%, age 40 ± 9 years, M:F 409:60) use NIV in their clinical practice. NIV was most exclusively being used in the ICU setting (68.4%) and the commonest indication for its use was chronic obstructive pulmonary disease (COPD) (71.4%). A significant number did not report use of a conventional ventilator for NIV support (62%). Oronasal mask was the overwhelming favorite among the sampled physicians (68.2%). In most of the cases, the treating physician initiated NIV (60.8%) and a baseline blood gas analysis was performed in only 71.1% of the cases (315/443). Nasal bridge pressure sores was the commonest complication (64.2%).

**Conclusions:**

NIV is being widely used in clinical practice in India for various indications. COPD is the most common indication for its deployment. There seems to be a marked variability in the patterns relating to actual deployment of NIV, including the site of initiation, protocols for initiation followed, and monitoring of patients.

## Introduction

Noninvasive ventilation (NIV) is a useful therapy for management of selected patients with respiratory failure.[1–4] Significant variability in the practice patterns of NIV use has been observed across the world.[5–7] However, NIV is of particular interest for a country like India for several reasons. India is a large country in which the healthcare system is facing major cost limitations. NIV has the potential to be a much cheaper option than conventional mechanical ventilation by reducing complications and length of hospital stay, and in some cases, by avoiding ICU admission when delivered outside the ICU. It can also be delivered by relatively simple equipments, reducing this part of the costs. Lastly, although there is lack of formal data, the diseases well treated by NIV, such as acute exacerbation of chronic obstructive pulmonary disease (COPD) and acute cardiogenic pulmonary edema have a high incidence in India and represent huge number of cases. The use of NIV may, however, be limited by different reasons including physician's training. It is therefore relevant to assess the current practice patterns of NIV use by the Indian physicians.

## Materials and Methods

### Instrument

A questionnaire that could capture the desired information was developed indigenously. Specific questions were developed based on the earlier surveys and perceived areas of interest. The questions revolved around the profile of the physician including the demographic details, specialty, place of work, whether they have used NIV and, if not, the reason for it. Physicians who responded in positive to the use of NIV, were directed to further questions that included duration, site and indications of NIV use, type of equipments utilized, and patterns of their use. Further questions regarding the infrastructure in place and the specifics related to provision of NIV were posed to the respondents. Finally, the physicians were asked about the adverse effects seen and common causes of failure of NIV in their experience. Responses were mostly objective and closed to facilitate analysis, ensure reproducibility, and enhance response rates. The questionnaire was initially sent to 10 residents and physicians, and the responses evaluated to ensure that the questionnaire was clear and consistent with no ambiguity. The questionnaire as well as the study methodology was approved by the Institutional Ethics Committee.

### Subjects

Around three thousand physicians from all over India who are life members of the Indian Society of Critical Care Medicine (ISCCM) and the National College of Chest Physicians (NCCP) of India, were the target population. The members of these two societies were from variable national geographical backgrounds and specialties and included Intensivists, Anesthesiologists, Pulmonologists, and General Internists. These distinctions were made based on the training that they had received.

Questionnaires were mailed along with a self-addressed envelope, to all the members on the addresses available in the directories over the next two months. The responses were awaited for next six months before E-mail reminders were sent to physicians whose E-mail ID's were available in the directories. At the end of a total eight months after mailing all the questionnaires, data were entered into a Microsoft excel worksheet and analysis conducted.

### Statistical analysis

Data was described using mean with standard deviation for continuous variables and as proportions for categorical variables. Various comparisons were carried out using independent *t*-test for continuous variables and chi-square test for categorical variables. Significance was considered at *P* < 0.05 (two tailed). SPSS version 11.0 (SPSS Inc. Chicago, IL, USA) was used for statistical analysis.

## Results

**Figure 1:**
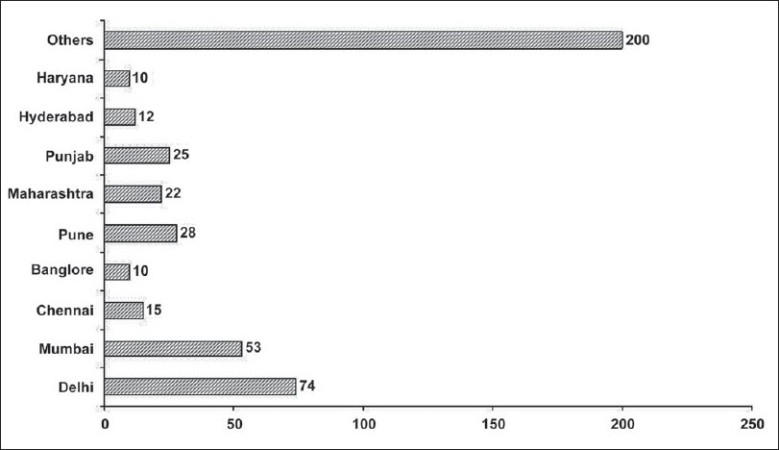
Geographic spread of respondents in the survey

Responses were received from 648 physicians (response rate: 21.6%). All respondents, however, did not reply to all the questions and the number of replies varied for each question. A majority of respondents comprised of Intensivists (63.2%). Geographically, almost all the regions of the country were represented in the survey sample with Delhi (*n* = 74, 16.5%) and Mumbai (*n* = 53, 11.8%) being the top two in terms of number of respondents ([Figure 1], Electronic Supplementary Material).

### Use and nonuse of NIV

**Figure 2:**
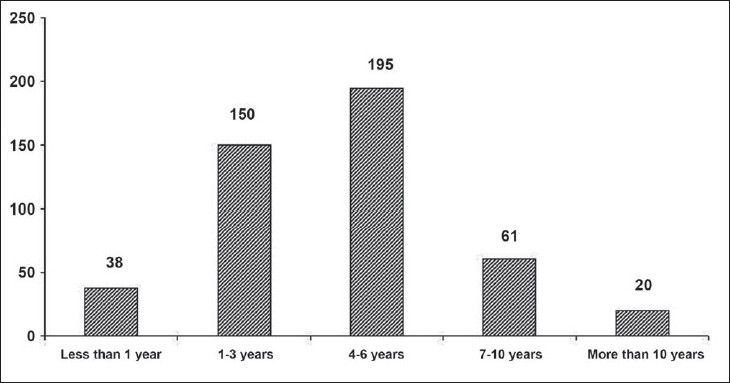
Number of years of NIV use for the study respondents

A large majority of physicians, *n* = 469, 72.4%; 40 ± 9 years; M:F 409:60, reported use of NIV in their practice, while the rest did not, *n* = 179, 27.6%; 50 ± 12 years; M:F 161:18. Respondents who were not using NIV in their clinical practice were likely to be older and in clinical practice for number of years [Table 1]. Moreover, Internists as a specialty and physicians working in smaller peripheral hospitals were also less likely to use NIV [Table 1]. Lack of experience with the modality was cited as the commonest reason for not using NIV in the clinical practice (47.8%) followed by lack of finances for setting up the facility (14.6%). Only five (2.8%) respondents were doubtful about the efficacy of NIV as a ventilatory strategy. As many as 19 respondents (10%) were not aware of this modality. The number of years of NIV use for the study respondents is shown in Figure 2.

**Table 1:** Comparative profile of physicians who reported noninvasive ventilation use and those who did not

	Use NIV in clinical practice (n = 469)	Do not use NIV in clinical practice (n = 179)	*P*
Age	39.6 years	48.7 years	<0.001
Gender (Males)	87.8	92.2	NS
Years since graduation			<0.001
0–5	75 (16.3)	9 (5.1)	
6–10	110 (23.9)	11 (6.2)	
11–15	115 (24.9)	33 (18.5)	
>15	161 (34.9)	125 (70.2)	
Specialty			<0.001
Intensivist	172 (37.0)	5 (2.8)	
Internist	58 (12.5)	39 (21.9)	
Respiratory Physician	100 (21.5)	67 (37.6)	
Anesthesiologist	122 (26.2)	25 (14.0)	
Place of work			<0.001
Super specialty hospitals	263 (56.6)	47 (27.6)	
Multispecialty hospitals with	142 (30.5)	47 (27.6)	
>100 beds	60 (12.9)	76 (44.7)	
Peripheral hospitals with < 100 beds			

Figures are in parentheses are in percentage

### Indications for NIV

**Figure 3:**
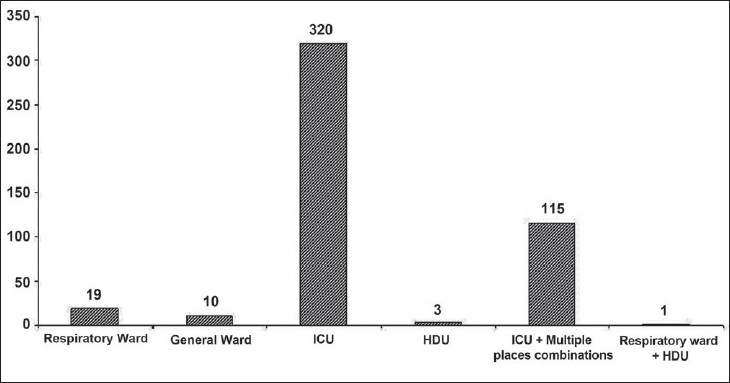
Common sites for NIV use in India ICU, Intensive care unit; HDU, high dependency unit)

**Figure 4:**
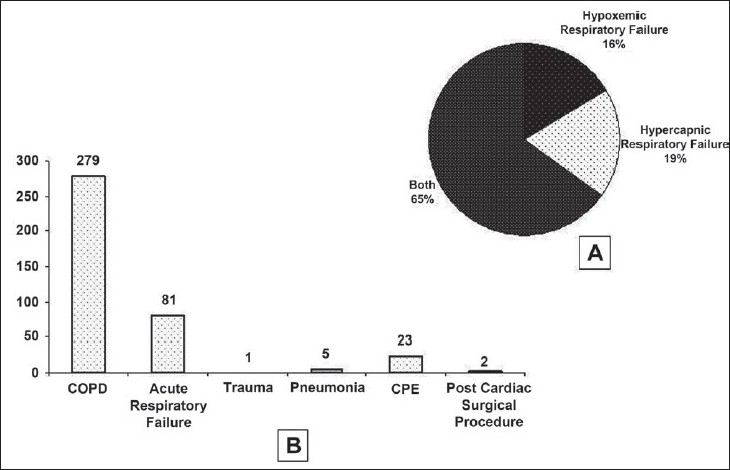
Indications for which of NIV is employed by the Indian physicians in terms of type of respiratory failure (A) as well as specific medical conditions (B)

Among the groups of respondents who were using NIV, the largest group was of the Intensivists (*n* = 172, 37%) followed by Anaesthesiologists (*n* = 122, 26.2%), Respiratory Physicians (*n* = 100, 21.5%), and General Internists (*n* = 58, 12.5%). Whereas, majority of intensivists are backed by institutional training in the provision of NIV (53.2%), others are not (Anaesthesiologists, 33%; Respiratory Physicians, 38.1%; General Internists, 22.8%; *P* < 0001). Majority were using NIV exclusively in the ICU (68.4%) and only a fraction of physicians were using NIV in general wards or respiratory wards [Figure 3]. Among the indications for NIV, a majority of respondents reported use both in hypoxemic and hypercapnic respiratory failure (64.9%), whereas COPD [Figure 3A] was the most common indication for hypercapnic respiratory failure, but there was no clear favoured indication for hypoxemic respiratory failure. Frequency of NIV use in specific indications under the categories of both hypoxemic and hypercapnic respiratory failure is presented in Table 2. Overall, COPD was reported as the most common indication by an overwhelming majority of respondents (71.4%). However, a significant number of respondents reported acute respiratory failure (ARF) secondary to other causes as the most common indication for NIV use in their clinical practice [Figure 4]. The number of years of NIV use by the physician did not have an impact on the indications for which NIV was employed.

**Table 2:** Frequency of noninvasive ventilation use in specific indications

	Number of respondents	Most often (%)	Often (%)	Rare (%)
Hypercapnic respiratory failure				
COPD	439	328 (74.7)	100 (22.8)	11 (2.5)
Neuromuscular disorder	334	23 (6.9)	137 (41.0)	174 (52.1)
Obesity Hypoventilation syndrome	392	102 (26.0)	196 (50.0)	94 (24.0)
Weaning in COPD	377	130 (34.5)	130 (34.5)	117 (31.0 )
Hypercapnic Cardiogenic pulmonary edema	376	98 (26.0)	124 (33.0)	154 (41.0)
Asthma	386	47 (12.2)	124 (32.1)	215 (55.7)
Hypoxemic respiratory failure	380	108 (28.4)	122 (32.1)	150 (39.5)
Cardiogenic pulmonary edema				
Pneumonia	362	41 (11.3)	175 (48.3)	146 (40.3)
Trauma	319	12 (3.8)	114 (35.7)	193 (60.5)
Post extubation respiratory failure	372	68 (18.3)	149 (40.1)	155 (41.7)
Acute Respiratory Distress Syndrome	354	32 (9.0)	106 (29.9)	216 ((61.0)
Weaning in other conditions	380	85 (22.4)	151 (39.7)	144 (37.9)

Most often, 5–10 patients in a month; often, 1–4 patients in a month; Rare, <1 in a month

### Equipment for provision of NIV

Most Indian physicians use portable pressure ventilators for application of NIV. A significant number of physicians do not use conventional ventilators for providing NIV support (62%). However, more recent users of NIV were more likely to be using conventional ventilators as compared to those who have been using NIV for a long time (87/186 for NIV use ≤3 years vs. 88/274 for NIV use ≥4 years, OR, 95%CI: 1.86, 1.26–2.73; *P* = 0.001). Also, General Internists in comparison to others as a specialty, were more likely to use conventional ventilators for providing NIV (34/58 vs. 141/402, OR, 95%CI: 2.31, 1.42–3.76; *P* = 0.001). Oronasal masks were the overwhelming favourite among the sampled physicians with as many as 68.2% reporting to its exclusive use. Another 26.0% physicians reported using nasal mask in combination with the oronasal mask, whereas only 5.1% used nasal mask exclusively. A majority of respondents preferred using the reusable mask (72.4%). Cidex (44.2%) and detergent with warm water (31.9%) are commonly used for sterilization of the mask.

### Provision of NIV and blood gas analysis

More than 15% physicians use NIV in the absence of availability of an arterial blood gas analysis machine, and Internists are most likely to practise this (20/56, 35.7%). Moreover, only 71.1% of the physicians were routinely performing a baseline blood gas analysis before initiation of NIV, whereas the others initiated it only on the basis of clinical judgment (28.9%). A repeat blood gas analysis within 4 hours was reported to be performed by less than half of the respondents (48.5%) and another 30% were doing a blood gas analysis in 4–6 hours time. NIV was reported to be mostly initiated by the attending physician himself (273/449, 60.8%) or the resident (127/449, 27.8%). The most common range of pressure at initiation of the NIV was 8–11 cm of H_2_O for IPAP and 4–6 cm of H_2_O for EPAP. Other ranges of pressures used in general as well as for COPD alone are presented in Table 3.

**Table 3:** Range of pressures used for provision of noninvasive ventilation

Pressure range (general)	Total respondents (general)	Number of respondents using the pressure range	Percent	Pressure range (COPD)	Total respondents	Number of respondents using the pressure range	Percent
IPAP						
4–7	443	49	11.1	8–12	440	165	37.5
8–11		265	59.8	13–16		233	53.0
12–15		122	27.5	17–20		36	8.2
16–19		7	1.6	21–24		6	1.4
EPAP						
1–3	441	91	20.6	<4	439	33	7.5
4–6		316	71.7	4–6		302	68.8
7–9		24	5.4	7–8		90	20.5
10–12		10	2.3	9–10		9	2.1
				>10		5	1.1

### Complications of NIV

**Figure 5:**
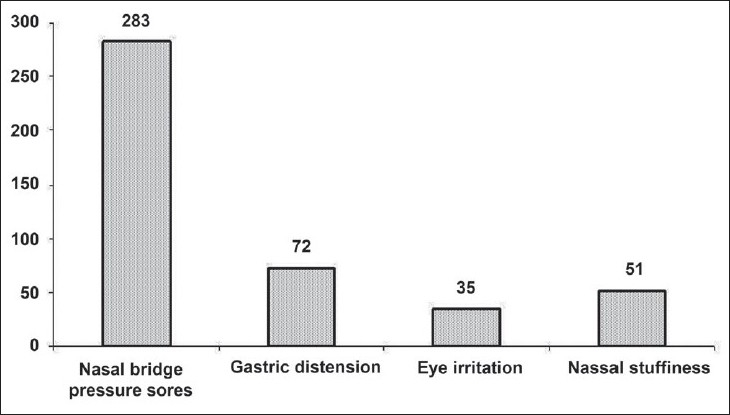
Frequency of the most common side effects of NIV use seen by the physicians

Nasal bridge pressure sore was by far the commonest complication seen (64.2%) by the physicians. Gastric distention (16.3%) and nasal stuffiness (11.6%) were the other side effects ([Figure 5], Electronic Supplementary Material). Increasing severity of illness (45.3%) and inability of the patient to cooperate and tolerate NIV (62.5%) were reported as the two common reasons for failure of NIV. Air leak was also common (26.6%). New NIV operator or user (6.4%) and unsupportive staff/nursing (10.4%) were rarely responsible for failure of NIV.

An overwhelming majority of physicians felt that guidelines regarding use of NIV would be useful (94%), only 31.6% of the respondents were aware about the existence of any guidelines. The awareness was highest among the Intensivists (37.7%) followed by Anaesthesiologists (34.5%).

## Discussion

The present study aimed to evaluate the practice patterns of NIV use among physicians from India. To the best of our knowledge, this is the first study of its kind in India although similar work has been carried out in the West.[8,9] The survey utilized a simple instrument that was developed indigenously. The questionnaire was kept simple, short, and concise with objective responses to maximize the response rate.

Although frequently observed in this kind of survey, the response rate to the survey was low, which was probably influenced by the survey methodology that was employed. Though almost 3000 questionnaires were mailed, it cannot be ascertained with certainty how many physicians actually received the same as many members do not update their change of addresses in the society directories. Given this scenario, the true response rate (given a different denominator of physicians who actually received the questionnaire) of the current study would be higher. Moreover, the average response rate in physician postal surveys has been determined to be just more than 50%.[10,11] It must be acknowledged though that a selection bias favoring the physicians who use NIV to respond may tend to occur. It is therefore difficult to extrapolate the specific figure of proportion of physicians using NIV to the whole of country.

The overall profile of the group of physicians that did not use NIV in their clinical practice was understandably different from those that did use NIV. It is likely that older physicians who work in peripheral hospitals and do not have subspecialty training, would be the group more likely to persist with traditional management approaches. Interestingly, the association between fewer years in clinical practice and NIV use was also found in the Ontario survey.[9]

One of the key findings of the study was the finding of marked underutilization of NIV outside ICU. Whereas NIV continues to be largely used in an intensive care setting, it is being increasingly deployed in general wards as well. The study by Plant and coworkers[12] where equivocal results were demonstrated with use on NIV in general wards has been largely responsible for this development. However, the same does not seem to be the case in the present survey and this is one area where awareness levels need to go up. This is especially important for resources-limited settings such as those in India where a perennial shortage of intensive care beds exists.

The indications for the use of NIV remained largely similar and did not differ by the place of use, hospital setup, physician specialty, or the number of years of use of NIV. The earlier two surveys also found COPD as the commonest indication for provision of NIV support.[8,9] This is pretty much on expected lines, given the weight of the evidence supporting the role of NIV in management of patients with exacerbation of COPD. However, very few respondents reported use of NIV for cardiogenic pulmonary edema and awareness regarding the use of NIV in this setting should also go up.

It was intriguing to note that despite broad consistencies in the indications for which NIV is used, significant heterogeneity was the norm when it came to the delivery techniques. Use of conventional ICU ventilators for provision of NIV was noted more frequently with Internists as a specialty and with those who were using NIV for lesser number of years. It appears that some of the more experienced and subspecialist operators are reluctant to use conventional ventilators for provision of NIV. Unless the same is the result of unavailability of conventional ventilators in the setup, one would wish that physicians attempt to maximize the utilization of available resources in the setup.

As per the current guidelines, most of the settings where NIV is used, backup conventional ventilators would be available,[13,14] and so was the case in the current survey where an overwhelming majority of physicians reported availability of backup conventional ventilators (92.2%). However, when it came to carrying out blood gas analysis before initiation of NIV, almost 30% did not report following this protocol and would go by their clinical judgment while initiating a patient on NIV. Moreover, 15% of physicians were actually using NIV in absence of availability of blood gas analysis in their setups. Although, we did not compare the outcomes of the patients that are managed in this fashion with those managed as per the standard protocols, these findings do lend credence to the feasibility of such an approach. These possibilities need to be explored in future studies, especially in resources-limited settings. It is well known that many of the healthcare setups in the peripheries of the country cannot offer anything more than oxygen therapy in terms of respiratory support to the patients. It may also be possible in many of these centers to procure a NIV but are probably discouraged because of the norm that NIV should be used only in monitored settings and with availability of backup conventional ventilators. However, in a setup where no other means of providing assisted ventilation is available, patients could be offered NIV as a stand-alone modality of assisted ventilation. Whereas this may not be done when a referral of the patient to a higher level of care is possible, it can certainly help to improve outcomes when no other option is available.

It was also noted that majority of times NIV was initiated by the physicians themselves. However, in many settings it is possible that physicians may not be available all the times and during such times the “window of opportunity” of initiating NIV in a timely fashion must not be missed. It is therefore worthwhile to train more nursing and paramedical staff in use of NIV.

It is concluded that a majority of participating physicians across different specialties, backgrounds, and from different healthcare setups use NIV in their clinical practice. The indications for which NIV is utilized were also similar across the study cohort. However, significant heterogeneity was seen in terms of training received and the delivery methods employed by the physicians from different specialties.
